# Quantitative Analysis of Electroplated Nickel Coating on Hard Metal

**DOI:** 10.1155/2013/631936

**Published:** 2013-08-06

**Authors:** Hassan A. Wahab, M. Y. Noordin, S. Izman, Denni Kurniawan

**Affiliations:** Faculty of Mechanical Engineering, Universiti Teknologi Malaysia, 81310 Skudai, Malaysia

## Abstract

Electroplated nickel coating on cemented carbide is a potential pretreatment technique for providing an interlayer prior to diamond deposition on the hard metal substrate. The electroplated nickel coating is expected to be of high quality, for example, indicated by having adequate thickness and uniformity. Electroplating parameters should be set accordingly for this purpose. In this study, the gap distances between the electrodes and duration of electroplating process are the investigated variables. Their effect on the coating thickness and uniformity was analyzed and quantified using design of experiment. The nickel deposition was carried out by electroplating in a standard Watt's solution keeping other plating parameters (current: 0.1 Amp, electric potential: 1.0 V, and pH: 3.5) constant. The gap distance between anode and cathode varied at 5, 10, and 15 mm, while the plating time was 10, 20, and 30 minutes. Coating thickness was found to be proportional to the plating time and inversely proportional to the electrode gap distance, while the uniformity tends to improve at a large electrode gap. Empirical models of both coating thickness and uniformity were developed within the ranges of the gap distance and plating time settings, and an optimized solution was determined using these models.

## 1. Introduction

Nickel coating is widely used for decorative and functional applications, by improving corrosion resistance, wear resistance, or by modifying other properties of the coated articles [1]. With tungsten carbide/cobalt (WC-Co) cemented carbides, nickel coating is explored as well for the purpose of jointing these hard metals with other substances, both softer and harder ones. Cemented carbides have high hardness and strength contributed to the tungsten carbide with some toughness contributed to the cobalt binder. These properties make them suitable as fortifying coating for metallic substrates for some applications. This is where brazing using electroplated nickel becomes a potential jointing technique. Chen et al. [2] jointed WC-Co to stainless steel by a brazing process using nickel interlayer between the two metals and found that fracture of the brazed joints occurred in the bulk WC-Co substrates, indicating the success of the jointing nickel interlayer.

In other applications requiring extreme wear resistance, cemented carbide properties are still insufficient, and coating it with harder and stronger substances such as diamond or diamond-like carbon is applicable. Electroplated nickel interlayer is explored for this purpose where adhesion between the two substances is the main issue. Common diamond coating technique, that is, chemical vapor deposition, requires high temperature in which the cobalt binder within cemented carbide favors reaction towards graphitic phase, an unexpected interlayer during diamond deposition process which leads to deleterious effect to the diamond coating [3, 4]. There is also problems related to residual stress at the interface caused by mismatch in thermal expansion coefficients between the two substances. These issues make pretreatments on the hard metal substrate prior to diamond deposition required. Introducing intermediate layer between the cemented carbide substrate and the diamond coating is a way for this purpose. The use of electroplated nickel as the interlayer is of interest in this study considering nickel's thermal expansion coefficient which is close to that of hard metal, and that diamond can deposit and grow on nickel substrate [3, 5, 6].

Electroplating for nickel deposition uses an electrolytic path. This method has advantages of having low reaction temperature which avoids residual stress caused by mismatch in thermal expansion coefficient, being economically viable, and being easy to control by manipulating the deposition parameters [7–9]. Thickness and uniformity are some of quality measures of electroplating results. This study attempts to improve the quality of electroplated nickel on hard metal substrate by selecting proper deposition parameters combination. Intensity of electric field and electrolyte resistance between anode and cathode are affected by the gap between electrodes [10]. Also, considering kinetics of reaction, electroplating is affected by the duration of reaction. Accordingly, in this study the electroplating time and the gap between electrodes varied, and their influence on the quality of deposited nickel layer is quantified by empirical models using design of experiment (DOE) so that the result could be objectively analyzed [11].

## 2. Experimental

### 2.1. Sample Preparation

A WC-6%Co rod of size 5 mm diameter × 150 mm length was cut using a precision cutter machine into moderate thin samples. The surface roughness of the samples was modified to *Ra* = 0.3 *μ*m by sand blasting using SiC with 180 *μ*m grit size. After blasting, these samples were ultrasonically cleaned with acetone for 20 minutes to remove any residuals, followed by rinsing with deionized water. Finally, the samples were cleaned by steam jet for 30 seconds to remove any remaining residuals.

### 2.2. Plating Setup

**Figure 1:**
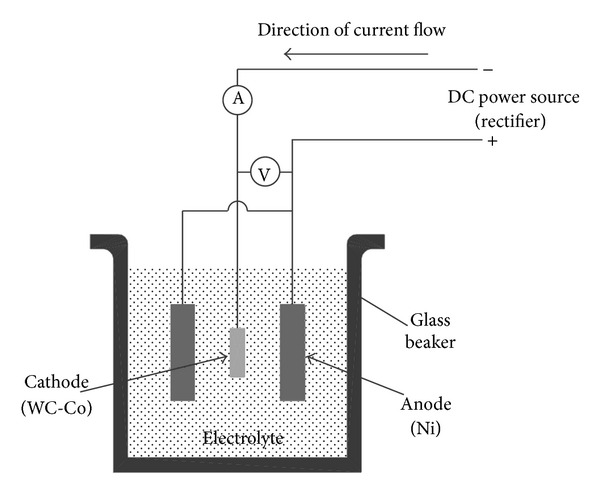
Schematic of the electroplating cell.

For the nickel deposition, an electroplating solution containing 400 g/L NiSO_4_·6H_2_O, 30 g/L NiCl_2_·6H_2_O, and 30 g/L H_3_BO_4_ was used. The pH level of the electrolyte solution was regulated to a value of 3.5 by adding 10% of dilute sulfuric acid to the electrolyte. The experiments were carried out in a bath with a temperature of 55°C and 1.0 V electric potential to produce 0.1 Amp of current under continuous agitation by a magnetic stirrer. Two pure nickel round plates of 20 mm diameter × 3 mm thickness were used as anodes. The cathode was the thin sample previously cut (5 mm diameter × 2.05 mm thick) WC-6%Co substrate, which was fixed at distinct distances from each anode.  Figure 1 illustrates the electroplating configuration and setup. The electrodeposition of nickel onto the substrate surface was performed at different holding times.

### 2.3. Characterization

Electroplated samples were mounted with resin and then sectioned by precision cutter for metallographic tests. The nickel coating thickness and interface were characterized by scanning electron microscope (SEM). Value of coating thickness is obtained from the average of three measurements on each SEM image.

The layer thickness variation (*k*) between the substrate face and its circumference was calculated using (1) as follows:

(1) k=[(coating  thickness  on⁡  surface   −coating  thickness  on⁡  circumference)   ×(coating  thickness  on⁡  surface)−1]×100%.

### 2.4. Deposition Parameters

Electrode gap and plating time were the input variables, termed in coded form as *x*_1_ and *x*_2_, respectively. The upper and lower limit values of the input variables were chosen in order to maintain interlayer thickness greater than 2 *μ*m with adequate quality. The selected upper and lower limit values for the electrode gap were 5 mm and 15 mm, respectively, and for the plating time the values were 10 min and 30 min, respectively. The middle limit values were 10 mm for the electrodes gap and 20 min for the plating time.

Design of experiments and results analysis were performed using commercial statistical analysis software (Design Expert 6.0 of Stat-Ease Inc.). A three-level factorial design with two variables as the input was employed [11]. The total treatment combinations required for the design were 9 combinations (Table 1).

For determining the relationship between the input and response, the collected data were analyzed using regression. In regression model, the empirical variable (response) is approximated based on a functional relationship between the estimated variable, *y*, and one or more input variables, *x*_1_ and/or *x*_2_. The model equation containing the input variables was fitted by using least square technique. Accordingly, the residual error measured by the sum of square deviations between the actual and the estimated responses was minimized. This involved the calculation of estimates for the regression coefficients, that is, the coefficients of the model variables including the intercept or constant term for statistical significance test.

## 3. Result and Discussion

**Figure 2:**
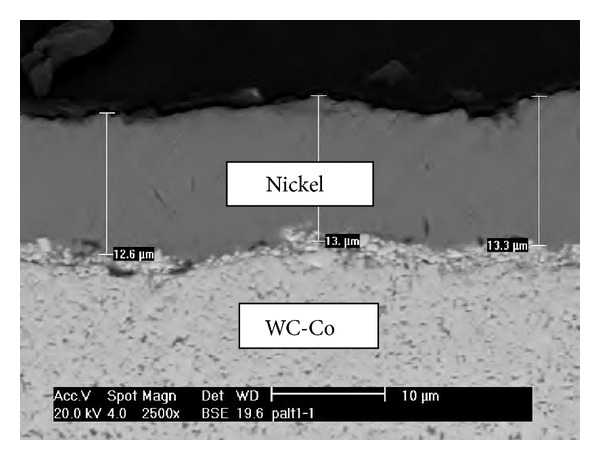
SEM image of electroplated nickel coating on hard metal surface when plating time is 10 min and anode/cathode gap distance is 5 mm.

Typical result of electroplated nickel coating on hard metal surface is as shown in Figure 2. The thickness and uniformity of the electroplated nickel coating were indeed affected by the plating time and electrode gap distance as presented in following subsections.

### 3.1. Coating Thickness

**Figure 3:**
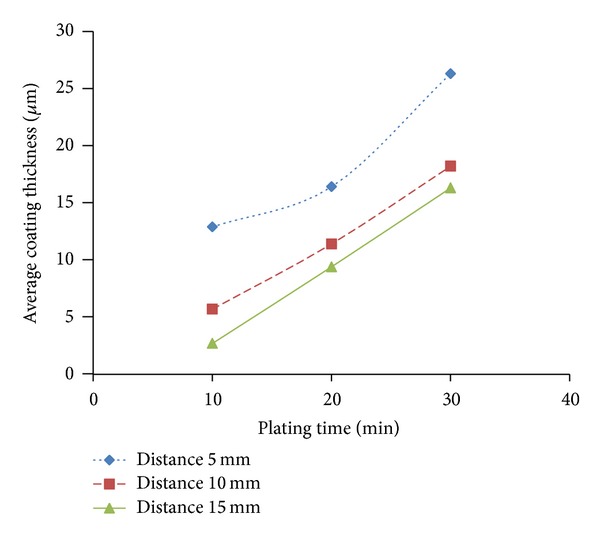
Average coating thickness versus plating time for three different anode/cathode gaps (pH: 3.5, current: 0.1 A, and voltage: 1.0 V).

The experimental results for coating thickness are shown in Figure 3. The electroplated nickel coating thickness is inversely proportional to the gap between the electrodes and is proportional to the deposition time. The highest coating thickness of 26.3 *μ*m was obtained at electrode gap 5 mm and deposition time of 30 minutes, while the lowest coating thickness of 2.7 *μ*m was resulted when the electrode gap is 15 mm and deposition time is 10 minutes.

To develop the empirical model of the coating thickness, a fit summary output to determine the most suitable regression model was evaluated. Linear model was selected for having the least probabilistic value, Prob > *F*, for representing coating thickness results. Analysis of variance was performed to test the significance of the regression model and its coefficients, with probabilistic value of less than 0.05 indicating significance. The linear model and its coefficients have a probabilistic value of well below 0.05 (Table 2). Furthermore, its coefficient of determination, *R* ^2^, of 0.96 is very close to unity, also indicating that the model closely approximates the coating thickness data. The model's adequate precision ratio of 22.6, which compares the range of the predicted values at the design points to the average prediction error, is well beyond the minimum adequacy limit of 4.

**Figure 4:**
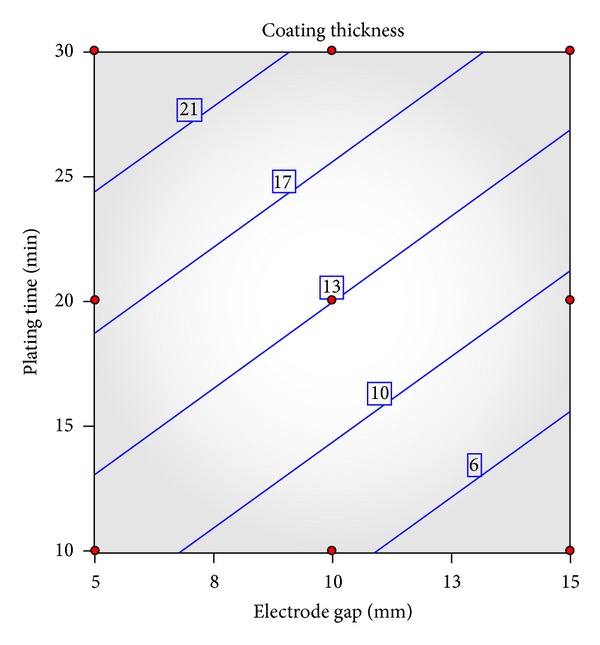
Response surface graph of contours for coating thickness.

The empirical model equation which relates between resulted coating thickness and the setting of gap distance and plating time is

(2) $$\begin{array}{c} T=9.16-0.91d+0.66t, \end{array}$$

where *T* is coating thickness, *d* is gap distance between electrodes (in mm), and *t* is plating time (in minutes). The model equation can be depicted in a contour graph (Figure 4). The developed model's predicted coating thickness for any combination of gap distance and plating time was confirmed within the range of 95% confidence interval. This verifies that the model is sufficient to represent the coating thickness data for this particular electroplated nickel deposition.

The finding that electrode gap distance is inversely proportional to coating thickness is in line with previous reports which found that when gap between electrodes is narrow the intensity of electric field increases and electrolyte resistance between anode and cathode decrease [10, 12]. These result in increasing metal deposition rate or thicker coating within a certain period of time. This study adds that the relation in particular settings is linear. Regarding the linear effect of deposition time to coating thickness, it is in agreement with Faraday's law in which deposition rate (or in this case the coating thickness resulted within a certain period) is proportional to the plating current and time [13, 14].

### 3.2. Layer Uniformity

**Figure 5:**
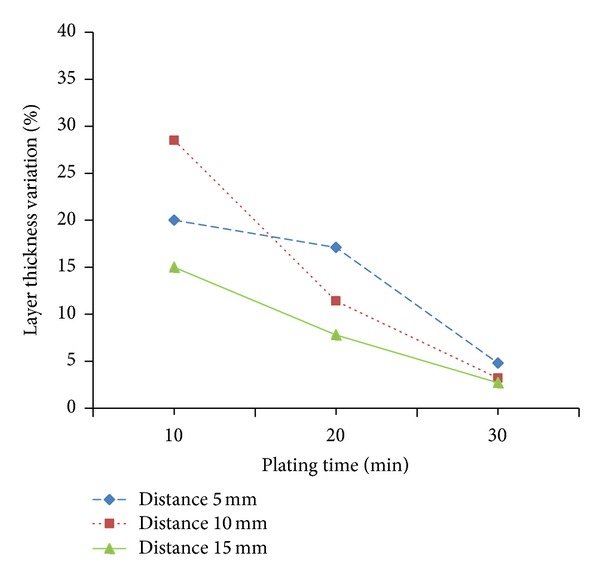
Thickness uniformity versus plating time at various electrode gaps.

The layer thickness variations between the substrate face and its circumference are shown in Figure 5. The combination between the electrode gaps and plating time used in this study can generate coating layer with up to a maximum of 26.5% thickness variation. The increase of both electrode gap and plating time tend to produce more uniform nickel coating on the hard metal substrate.

In developing the empirical model for coating thickness uniformity as function of gap distance and plating time, similar steps used to develop model for coating thickness was employed. A preliminary diagnosis was conducted to determine the appropriate power transformation for the model. This was assessed by diagnosing Box-Cox plot of coating thickness uniformity. The lowest point of the plot that might result in the minimum residual sum of square in the transformed model (*λ*) was 0.61. Accordingly, a logarithmic transformation (*λ* = 0) was selected as the closest value to the actual one and as the most appropriate power transformation. Next step was determining the suitable regression model. The probabilistic value, Prob > *F*, was calculated, and linear model was selected for having the least probabilistic value. Then, analysis of the variance was performed in order to test the significance of the selected regression model and its coefficients (Table 3). In the same way as before, the maximum probabilistic value of 5% was set for the model, and its coefficients were to be considered significant.

The empirical model equation which relates coating thickness uniformity (*k*) to gap distance (*d*) and plating time (*t*) was found to be

(3) $$\begin{array}{c} \ln k=4.48-0.05d-0.08t. \end{array}$$

**Figure 6:**
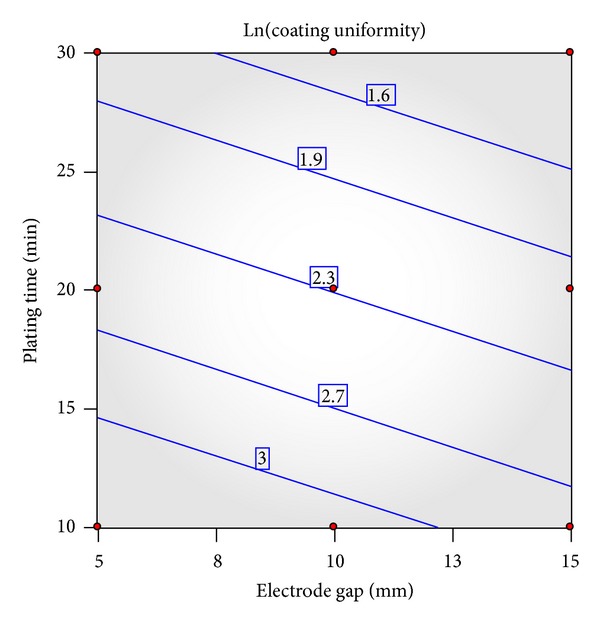
Response surface graph of contours for surface roughness.

This model can be represented by the graph of contours (Figure 6). The model's high coefficient of determination (*R* ^2^ = 0.92) and adequate precision (of 14.56) indicated that the model is adequately representative. At any combination of gap distance and plating time, the model's predicted coating thickness uniformity is within the range of 95% confidence interval. This verifies the adequacy represented by the model.

Due to the nature of the process, obtaining high degree of coating uniformity via electroplating technique is practically difficult. The ability of producing uniform coating thickness over a surface by electroplating process is associated with current distribution on that surface [1]. Current distribution is usually determined by the shape of the surface and its relative position with respect to the anode [15]. Edges and recesses on a surface often receive more current and this consequently results in thicker deposits on them [16]. Placing the electrodes at an adequate distance relative to one another might reduce the thickness variation of an electroplated coating by making the current distribution on the surface to be nearly uniform [10].

This study, in which 3% to 26.5% layer thickness variations were obtained, found that the nickel coating was more uniform when the hard metal substrate is placed at longer distance from the anodes. The result is in good agreement with a previous report that found that short electrode gap produces high offset voltage which causes larger current to be concentrated at the edges and the corner of the electrode [10]. Under this condition, reduction of reactant on the electrode surface is faster, and variation in reactants diffusion between the electrode edges and center region surface could increase [10]. When the distance between electrodes is increased the offset set voltage decreases and plating current lowers. Consequently, the current distribution at the surface area is improved [10].

Electroplating duration is also believed to have a positive effect in acquiring uniform distribution of the coating over the substrate surface. The result obtained from this study showed that plating time is significant in producing uniform coating thickness distribution. This could be explained by the fact that deposited atoms occupy the defect sites (edges, corners, steps, and kinks) on substrate surface after either direct or lateral diffusion. When atoms reach the defected sites they start to back themselves in the form of atoms lines across the substrate surface forming the crystalline structure of the deposit [17]. This action mainly depends on local lattice energy (incorporate more atoms to metal matrix at the cathode) and the plating time, as short time does not allow the completion of this deposition mechanism and could be the reason for nonuniform coating distribution.

### 3.3. Optimum Condition

Generally, nickel coating for industrial applications must be obtained with an acceptable degree of quality and uniformity. The empirical model developed here revealed the possibility of producing electroplated layer thickness with minimum degree of nonuniformity along the substrate surfaces by setting the electrode gap and plating time. In practice, it is difficult to obtain 100% uniform electroplated coating over a surface. However, low coating thickness variation across the surface is desired. 

**Figure 7:**
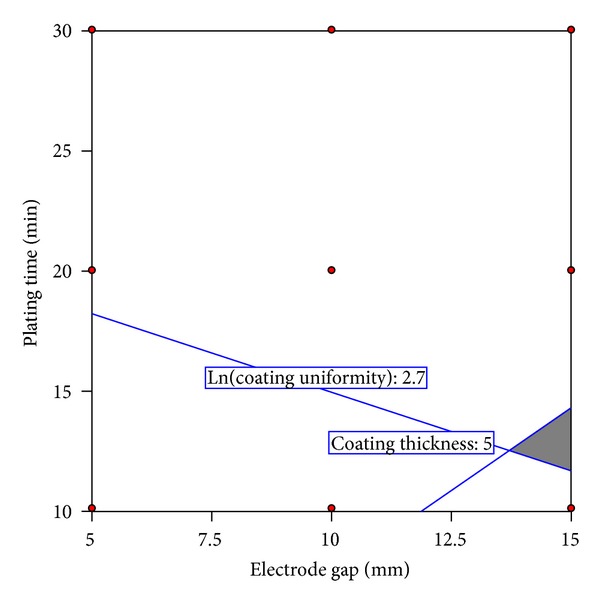
Overlay plot of the input variables for the predetermined output criteria of maximum of 5 *μ*m coating thickness and minimum of 15% coating uniformity. (The grey area is the optimized solution.)

This study, where the electroplated nickel is intended to be the interlayer between hard metal substrate and diamond coating, expects the nickel coating not to be excessive (low thickness) but with high uniformity. This can be achieved by selecting a suitable combination of electrodes gap and plating time. An optimized solution of these two variables should be determined. With the help of the empirical models developed previously, this optimized solution can be determined. The effect of each input variable (gap distance and plating time) on the electroplating responses (coating thickness and uniformity) is quantifiable using the models. It is presumed that coating thickness of 5 *μ*m is adequate to withstand cracks or delamination when the hard metal is subjected to heat treatment and diamond deposition. Also the deposited coating should have thickness uniformity of no less than 15% because below that point narrow or nonsolution domains might establish. Optimization of the responses is when the electrode gap and plating time combination is within the grey area of the overlay plot (Figure 7). The optimized solution is represented by the intersection between the solutions of coating thickness and the solutions for layer thickness variation.

## 4. Conclusion

The attempt to obtain electroplated nickel coating on WC-6%Co substrate was performed by varying electrode gap distance and plating time, with coating thickness and uniformity as the responses. Coating thickness is proportional to the plating time and inversely proportional to the electrode gap distance. The coating thickness uniformity tends to be better at a large electrode gap. Empirical models of both electroplating responses were developed within the ranges of the input variables. The models can determine the optimized solutions of gap distance and plating time combination for obtaining adequate coating thickness with high uniformity.

## Figures and Tables

**Table 1:** Design layout of experiment.

Standard	Distance (mm)	Time (min)	Coded form	
			*x*_1_	*x*_2_
1	15	20	1	0
2	10	20	0	0
3	15	10	1	−1
4	5	10	−1	−1
5	5	20	−1	0
6	10	10	0	−1
7	15	30	1	1
8	5	30	−1	1
9	10	30	0	−1

**Table 2:** Analysis of variance of the coating thickness model.

Source	Sum of square	Degree of freedom	Mean square	*F* value	Prob > *F*
Model	383.35	2	191.67	66.27	<0.05
Gap distance	123.10	1	123.10	42.63	<0.05
Plating time	260.04	1	260.04	89.91	<0.05
Residual	17.35	6	2.89		
Pure error		0			

Cor total	400.70	8			

**Table 3:** Analysis of variance for the coating thickness uniformity.

Source	Sum of square	Degree of freedom	Mean square	*F* value	Prob > *F*
Model	4.53	2	2.27	33.37	<0.05
Gap distance	0.43	1	0.43	6.36	0.04
Plating time	4.10	1	4.10	60.38	<0.05
Residual	0.41	6	0.07		
Pure error		0			

Cor total	4.94	8			
